# Polysomnographic Assessment of Sleep Comorbidities in Drug-Naïve Narcolepsy-Spectrum Disorders—A Japanese Cross-Sectional Study

**DOI:** 10.1371/journal.pone.0136988

**Published:** 2015-08-31

**Authors:** Taeko Sasai-Sakuma, Akihiko Kinoshita, Yuichi Inoue

**Affiliations:** 1 Department of Somnology, Tokyo Medical University, Tokyo, Japan; 2 Department of Life Sciences and Bio-informatics, Division of Biomedical Laboratory Sciences, Graduate School of Health Sciences, Tokyo Medical and Dental University, Tokyo, Japan; 3 Japan Somnology Center, Neuropsychiatric Research Institute, Tokyo, Japan; Osaka University, JAPAN

## Abstract

This is a large cross-sectional study which aimed to investigate comorbidity rate, degree of sleep-related breathing disorder, polysomnigraphically diagnosible rapid eye movement sleep behavior disorder/rapid eye movement sleep without atonia and periodic limb movements during sleep in Japanese drug-naïve patients with narcolepsy-spectrum disorders. A total of 158 consecutive drug naïve patients with narcolepsy with cataplexy, 295 patients with narcolepsy without cataplexy and 395 patients with idiopathic hypersomnia without long sleep time were enrolled. From retrospectively analyzed data of nocturnal polysomnography and multiple sleep latency test, higher rates of periodic limb movements during sleep (> = 15 h^-1^) (10.2%) and polysomnographically diagnosable rapid eye movement sleep behavior disorder (1.9%) were found in patients with narcolepsy with cataplexy. They had more severe periodic limb movements during sleep especially during rapid eye movement sleep and higher percentages of rapid eye movement sleep without atonia than the other two patient groups. In the present large sample study, Japanese drug naïve patients with narcolepsy with cataplexy showed the highest comorbidity rates of periodic limb movements during sleep, polysomnographically diagnosable rapid eye movement sleep behavior disorder and rapid eye movement sleep without atonia among those with the other narcolepsy-spectrum disorders; the rates were lower than those for Western patients.

## Introduction

Narcolepsy is characterized by excessive daytime sleepiness (EDS) and increased rapid eye movement (REM) sleep propensity leading to sleep onset REM sleep period (SOREMP), cataplexy (CA), sleep paralysis (SP), or hypnagogic hallucination (HH)[1]. The EDS and increased REM sleep propensity are known to be partly associated with the degree of loss of hypocretin-1 (orexin) producing neuron located in the hypothalamus[2]. The hypocretinergic dysfunction is highly specific to human leukocyte antigen (HLA)-DQB1*0602 positive NA with CA (NA-CA)[3, 4] but is observed in only 10–35% of patients with narcolepsy without cataplexy (NA w/o CA)[5]. In NA w/o CA, the rates of patients having the REM-related symptoms described above are also lower than in NA-CA and higher than in idiopathic hypersomnia without long sleep time (IHS w/o LST) depending on positivity/negativity of HLA- DQB1*0602[6, 7]. These findings are indicative of a disease continuity of NA and IHS w/o LST, so-called NA-spectrum disorders[6, 8].

Reports of the last decade have described motor dysregulation during sleep including periodic limb movements during sleep (PLMS)[9–12], REM sleep behavior disorder (RBD)[13–15], and REM sleep without atonia (RWA) as its physiological background[12–14, 16], and sleep-related breathing disorder (SRBD)[17–19] in patients with NA-CA. Regarding the mechanism of the motor dysregulation in NA-CA described above, the hypocretinergic dysfunction associated with abnormal dopaminergic function has been inferred to play a causative role[20, 21]. Hypocretinergic dysfunction has also been reported to be associated with SRBD in terms of deregulation of energy homeostasis[22] or attenuation of ventilatory responses[23]. However, few reports have described differences in the prevalence or polysomnographic characteristics of all the above mentioned comorbidities among NA-CA, NA w/o CA and IHS w/o LST in detail[16]. Moreover, no study has investigated the prevalence or degree of these sleep comorbidities in Asian patients with narcolepsy-spectrum disorders, although there are racial differences in the prevalence of NA, PLMS, RLS or SRBD[24–27].

The aim of this study was to assess nocturnal polysomnographic (n-PSG) findings of comorbid sleep disorders of SRBD, PLMS, and RBD/REM sleep without atonia [RWA] in a large drug-naïve Japanese sample of patients with NA-CA, NA w/o CA, and IHS w/o LST.

## Materials and Methods

### Subjects

The Ethical Committee of the Neuropsychiatric Research Institute approved this study. Written informed consent was obtained from all participants. Among the eligible consecutive patients with hypersomnia who visited the outpatient clinic of the Japan Somnology Center during May 2008 –February 2014, patients who met the following inclusion criteria were enrolled in this study: (1) diagnosed as NA-CA, NA w/o CA, or IHS w/o LST based on clinical symptoms and findings of a nocturnal polysomnography (n-PSG), and subsequent multiple sleep latency test (MSLT), according to the diagnostic criteria presented in the Second Edition of the International Classification of Sleep Disorders (ICSD-2); and (2) free of psychostimulant medication, antidepressants as anticataplectic drugs or hypnotics, which are potentially associated with SRBD, PLMS or REM sleep without atonia (RWA), when they underwent n-PSG and MSLT. During clinical interviews, board-certified sleep-disorder specialist physicians carefully confirmed the presence/absence of cataplexy characterized by sudden loss of bilateral muscle tone provoked by strong emotions such as laughter, anger, fear, and surprise. For all subjects, the Epworth Sleepiness Scale (ESS)[28] was self-checked at the first visit. Furthermore, their clinical charts were reviewed to investigate the self-reported existence of REM-related symptoms (SP and HH). The diagnostic criteria of narcolepsy-spectrum disorders were as follows; (1) NA-CA: EDS lasting for more than three months, shorter than eight minutes of mean sleep latency and twice or more SOREMPs on MSLT, or lower than 110pg/ml of hypocretin-1 in the cerebro-spinal fluid, and cataplexy, (2) NA w/o CA: EDS lasting for more than three months, and shorter than eight minutes of mean sleep latency and twice or more SOREMPs on MSLT, (3) IHS w/o LST: EDS lasting for more than three months, shorter than eight minutes of mean sleep latency and once or less SOREMPs on MSLT, and 6–10 total sleep time on n-PSG. Patients suspected of behavioral induced insufficient sleep syndrome or circadian rhythm sleep disorder from clinical interview underwent recording of their sleep-wake schedule by actigraphy or sleep logs. According to the ICSD-2[1], based on their clinical interview, actigraphy data or sleep logs, patients who had longer sleep durations on weekends than on weekdays, shorter sleep times during weekdays and daytime sleepiness were diagnosed as behavioral induced insufficient sleep syndrome. In addition, patients who seemed to have persistent or recurrent pattern of sleep disturbance due to alterations of the circadian timekeeping system or a misalignment between the timing of the individual’s circadian rhythm of sleep propensity and the 24- hour social and physical environments, were diagnosed as circadian rhythm sleep disorders. Then, patients diagnosed as these disorders were carefully excluded from analyses. Consequently, 158 patients with NA-CA, 295 patients with NA w/o CA and 395 patients with IHS w/o LST were recruited in this study.

### Recordings of n-PSG and MSLT

Diagnostic nocturnal video-PSGs were performed using a standard system (Alice 5; Philips Respironics) including six channels of scalp electroencephalographic (EEG) data (F3/A2, F4/A1, C3/A2, C4/A1, O1/A2, O2/A1), two electrooculographs, submental electromyography (EMG), electrocardiography, nasal/oral airflow data, a percutaneous oximetry sensor for recording oxygen saturation data, a microphone for detecting snoring sounds, chest/abdominal respiratory effort data, and bilateral anterior tibialis EMG. Sleep stages and EEG arousal, PLMS, respiratory events and RWA were scored according to the criteria set by American Academy of Sleep Medicine (AASM)[29]. REM sleep periods observed within 15 min from sleep onset on n-PSG were defined as SOREMPs.

The MSLT consist of five naps was performed according to the standard protocol using a standard system (Alice 5; Philips Respironics) including six channels of scalp electroencephalographic (EEG) data (C3/A2, C4/A1, O1/A2, O2/A1), two electrooculographs, submental electromyography (EMG) and electrocardiography[30, 31]. A sleep onset REM sleep period (SOREMP) was defined as the appearance of an epoch of REM sleep during the first 15 min of naps on the MSLT.

### Evaluation of RBD symptoms, REM sleep without atonia and PLMS on n-PSG

According to the ICSD-2[1], presence of abnormal REM sleep behaviors on nocturnal video-PSG was defined as clinical symptoms of RBD.

According to the aforementioned scoring rules[29], board-certified sleep technologists carefully scored RWA on the chin EMG. Referring to the recent study defining cut-off value of RWA to discriminate RBD from controls, subjects having more than 1.22% of a proportion of 3-sec mini epochs with phasic activity to the total duration of REM sleep were defined as those with RWA with allowance of scoring using 3-sec epoch alternative to 2-sec epoch[32].

Leg movements were defined as limb EMG activity with 0.5–10.0 s of duration having amplitude with an 8 μV increase in limb EMG voltage above resting EMG. A set of four or more consecutive leg movements with 5.0–90.0 s of intervals was defined as the PLMS series[29].

### Statistical analysis

To test the normality and equality of variances of the continuous variables, the Shapiro–Wilk test and Levene test were applied. After checking the normality and equality of variances with a *p* value greater than .05, a Kruskal–Wallis test was conducted, followed by a post-hoc Mann–Whitney U-test with Bonferroni correction (*p* <.05/3) to compare the continuous variables among the groups. Chi-square tests with rest error tests were also used to compare the categorical variables among the groups. The cut-off values of apnea–hypopnea index (AHI) and PLMS index were respectively set at 5/h and 15/h for diagnosis of SRBD and PLMS to compare the comorbidity rates of these disorders with the rates reported in the previous studies. All statistical analyses were conducted using software (SPSS 17.0; SPSS Inc.). Significance was inferred for *P* <.05.

## Results

### Clinical data and measures on n-PSG and MSLT

Clinical data and measures on n-PSG and MSLT are presented in Table 1. Patients with NA-CA showed higher BMI than those with NA w/o CA and IHS w/o LST (χ^2^ = 37.283, df = 2, *P* <.001). Rates of patients with SP and the rate with HH were higher in patients with NA-CA than in patients with NA w/o CA and IHS w/o LST (SP: χ^2^ = 69.160, df = 2, *P* <.001; HH: χ^2^ = 65.381, df = 2, *P* <.001). Regarding n-PSG measures, patients with NA-CA showed a higher arousal index (χ^2^ = 62.610, df = 2, *P* <.001), percentage of N1 (χ^2^ = 73.276, df = 2, *P* <.001) and rate of patients with SOREMP (χ^2^ = 183.917, df = 2, *P* <.001). They also showed lower sleep efficiency (χ^2^ = 46.225, df = 2, *P* <.001) and percentage of N2 (χ^2^ = 86.376, df = 2, *P* <.001) than patients with NA w/o CA and IHS w/o LST. Regarding the measures of MSLT, patients with NA-CA showed shorter mean sleep latency (χ^2^ = 172.507, df = 2, *P* <.001) and mean REM latency (χ^2^ = 91.175, df = 2, *P* <.001), and higher percentage of naps with SOREMP (χ^2^ = 681.870, df = 2, *P* <.001) than patients with NA w/o CA and IHS w/o LST.

**Table 1:** Clinical data and measures of nocturnal polysomnography and multiple sleep latency test of the three patient groups.

	NA-CA (*n* = 158)	NA w/o CA (*n* = 295)	IHS w/o LST (*n* = 395)	*P*	*ES* *
Sex, male: female	78:80	171:124*	192:203	.039	.09
Age at examination, yr	28.2±10.9	25.7±8.7^b)^	29.0±9.4	< .001	b) .18
ESS score	17.1±4.4	16.3±4.4	16.1±4.5	.063	
BMI, kg/m^2^	23.9±4.1 ^a)^ ^,^ ^b)^	21.7±2.9	21.9±3.5	< .001	a) .27, b) .24
Sleep paralysis, *n* (%)	103(65.2)*	93(31.5)	114(28.9)	< .001	.29
Hypnagogic hallucination, *n* (%)	111(70.3)*	123(41.7)	129(32.7)	< .001	.28
n-PSG measures					
total sleep time, min	501.7±56.4	501.3±65.4	495.9±56.8	.321	
sleep efficiency, %	89.4±8.0^a)^ ^,^ ^b)^	94.7±26.0	93.4±24.2	< .001	a) .31, b) .23
arousal index, h^-1^	16.7±14.2^a)^ ^,^ ^b)^	10.6±5.2	11.4±7.9	< .001	a) .35, b) .31
stage N1, %	14.2±8.3^a)^ ^,^ ^b)^	9.1±5.8	9.8±6.4	< .001	a) .38, b) .32
stage N2, %	44.0±12.9^a)^ ^,^ ^b)^	53.3±9.7	53.9±9.1	< .001	a) .37, b) .38
stage N3, %	7.5±6.9	8.3±6.9	7.4±6.9	.140	
stage REM, %	20.6±8.0	23.9±14.9	21.3±5.9	.110	
SOREMP, *n* (%)	80 (50.6)*	85 (28.8)	8 (2.0)	< .001	.47
MSLT measures					
mean sleep latency, min	2.0±1.9^a)^ ^,^ ^b)^	3.2±1.9	4.5±2.0	< .001	a) .33, b) .34
mean REM latency, min	3.2±2.4^a)^ ^,^ ^b)^	5.2±2.5	6.7±3.5	< .001	a) .38, b) .31
percentage of naps with SOREMP, %	81.8±22.0^a)^ ^,^ ^b)^	66.6±21.4^b)^	5.4±9.2	< .001	a) .34, b) .85

NA, narcolepsy; CA, cataplexy; IHS w/o LST, idiopathic hypersomnia without long sleep time; ESS, Epworth Sleepiness Scale; BMI, body mass index; n-PSG, nocturnal polysomnography; REM, rapid eye movement; SOREMP, sleep onset REM sleep period; MSLT, multiple sleep latency test; n.s., not significant. ES, effect size.

Values are expressed as mean±standard deviation.

Percentage of naps with SOREMP was calculated as follows; (the number of naps with SOREMP)/(the number of total naps)*100.

*Effect size was estimated by Cramer’s V for 2*3 Chi-square test and r for Kruskal-Wallis test (> = 0.10, small effect; > = 0.30, medium effect; > = 0.50, large effect).

^a)^ P< .01 versus NA w/o CA,

^b)^ P< .01 versus IHS w/o LST.

### Comorbidity rates of SRBD, PLMS, polysomnographically diagnosable RBD and RWA

Table 2 presents the rates of patients with SRBD, PLMS, *polysomnographically diagnosable RBD* and RWA in the three patient groups. No differences were found in the comorbidity rates of obstructive sleep apnea (OSA) or central sleep apnea (CSA) (5/h^-1^ and 15/h^-1^ of obstructive/ central AHI) among the three groups. The rate of patients having 5 h^-1^ or more of total PLMS index was higher in patients with NA-CA than in patients with NA w/o CA and IHS w/o LST (χ^2^ = 17.682, df = 2, *P*< .001). Patients with NA-CA also showed higher comorbidity rates of PLMS (5 h^-1^) during either NREM sleep (χ^2^ = 10.324, df = 2, *P* = .006) or REM sleep (χ^2^ = 6.486, df = 2, *P* = .039) than the other two patient groups. The rate of patients having 15 h^-1^ or more of total PLMS index was higher in patients with NA-CA compared with patients with NA w/o CA and IHS w/o LST (χ^2^ = 8.573, df = 2, *P* = .014). Patients with NA-CA also showed higher comorbidity rates of PLMS (15 h^-1^) during either NREM sleep (χ^2^ = 8.134, df = 2, *P* = .017) or REM sleep (χ^2^ = 8.031, df = 2, *P* = .018) than the other two patient groups. As for polysomnographically diagnosable RBD (manifestation of RBD and positive finding of RWA on n-PSG), patients with NA-CA also showed a higher comorbidity rate of polysomnographically diagnosable RBD (χ^2^ = 29.866, df = 2, *P* < .001). Patients with IHS w/o LST showed a lower rate of RWA compared with the other two narcoleptic groups (χ^2^ = 6.890, df = 2, *P* = .032).

**Table 2:** Comparisons of comorbidity rate of SRBD, PLMS, and RBD like symptoms and findings of RWA.

	NA-CA (*n* = 158)	NA w/o CA (*n* = 295)	IHS w/o LST (*n* = 395)	*P*	Cramer’s *V*
OSA (OAHI≥5/hr)	22 (14.4)	26 (8.9)	67 (17.0)	.069	
OSA (OAHI≥15/hr)	3 (1.9)	4 (1.4)	16 (4.1)	.083	
CSA (CAHI≥5/hr)	1 (0.6)	2 (0.7)	4 (1.0)	.565	
CSA (CAHI≥15/hr)	0 (0.0)	0 (0.0)	0 (0.0)	-	
PLMS (total PLMS index≥5/hr)	39 (24.7)*	31 (10.5)	52 (13.2)	< .001	.15
PLMS during NREM sleep (PLMS index during NREM sleep≥5/hr)	21 (13.3)*	14 (4.7)	36 (9.1)	.006	.11
PLMS during REM sleep (PLMS index during REM sleep≥5/hr)	12 (7.6)*	15 (5.1)	11 (2.8)	.039	.10
PLMS (total PLMS index≥15/hr)	16 (10.2)*	10 (3.4)	24 (6.1)	.014	.10
PLMS during NREM sleep (PLMS index during NREM sleep≥15/hr)	14 (8.9)*	8 (2.7)	23 (5.8)	.017	.10
PLMS during REM sleep (PLMS index during REM sleep≥15/hr)	7 (4.4)*	4 (1.4)	4 (1.0)	.018	.10
RWA	42 (26.6)	71 (24.1)	70 (17.7)*	.032	.09
polysomnographically diagnosable RBD ^a)^	3 (1.9)*	1 (0.3)	0 (0.0)	< .001	.19

NA, narcolepsy; CA, cataplexy; IHS w/o LST, idiopathic hypersomnia without long sleep time; OSA, obstructive sleep apnea; OAHI, obstructive apnea hypopnea index; CSA, central sleep apnea; CAHI, central apnea hypopnea index; PLMS, periodic limb movements during sleep; REM, rapid eye movement; RBD, REM sleep behavior disorder; RWA, REM sleep without atonia.

^a)^ abnormal REM sleep behaviors on nocturnal video-polysomnography and findings of RWA

Values are expressed as number of patients (%).

* *P*< .01 compared to values in the other groups

### Comparison of apnea–hypopnea index, PLMS index and percentages of phasic and tonic EMG activity

**Fig 1:**
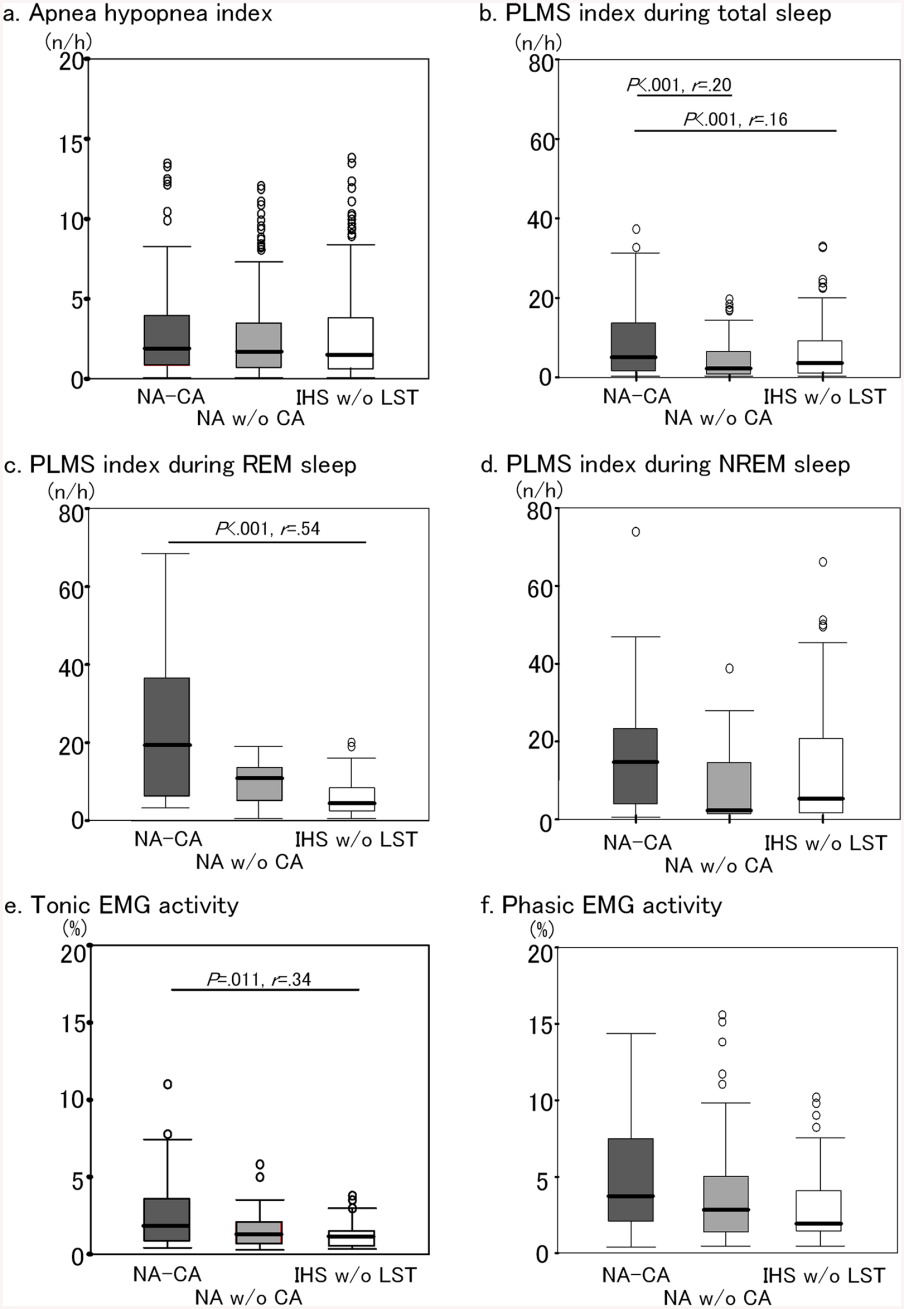
Comparisons of AHI, PLMS index and amount of RWA among the three patient groups. AHI, apnea hypopnea index; PLMS, periodic limb movements during sleep; RWA, rapid eye movement (REM) sleep without atonia; NA, narcolepsy; CA, cataplexy; IHS w/o LST, idiopathic hypersomnia without long sleep time; NREM, non-REM; EMG, electromyogram.

Fig 1a–1f presents the apnea–hypopnea index (AHI), PLMS index and percentages of phasic and tonic EMG activity in NA-CA, NA w/o CA, and IHS w/o LST. No differences were found in the apnea–hypopnea index among the three patient groups (mean±standard deviation was 3.3±4.5 h^-1^ in NA-CA, 2.7±3.5 h^-1^ in NA w/o CA and 3.8±7.2 h^-1^ in IHS w/o LST). Patients with NA-CA had higher PLMS index during total sleep than those with NA w/o CA and IHS w/o LST (χ^2^ = 19.692, df = 2, *P* <.001; 5.6±14.4 h^-1^ in NA-CA, 2.0±6.4 h^-1^ in NA w/o CA and 3.0±6.4 h^-1^ in IHS w/o LST). They also had higher PLMS index during REM sleep than those with IHS w/o LST (χ^2^ = 16.489, df = 2, *P* <.001; 7.0±14.5 h^-1^ in NA-CA, 4.5±9.3 h^-1^ in NA w/o CA and 2.7±6.9 h^-1^ in IHS w/o LST). In contrast, no differences were found in the PLMS index during NREM sleep among the three patient groups (14.2±19.9 h^-1^ in NA-CA, 7.3±15.5 h^-1^ in NA w/o CA and 11.7±20.1 h^-1^ in IHS w/o LST). Regarding RWA, patients with NA-CA had higher tonic EMG activity than those with IHS w/o LST (χ^2^ = 6.665, df = 2, *P* = .036; 2.8±2.8% in NA-CA, 1.9±1.9% in NA w/o CA and 1.1±0.9% in IHS w/o LST). The phasic EMG activity showed no differences among the three groups (5.6±5.5% in NA-CA, 4.4±4.6% in NA w/o CA and 2.7±3.3% in IHS w/o LST) (Fig 1).

## Discussion

The NA-CA group exhibited (1) higher REM sleep propensity manifested as higher percentage of naps with SOREMPs and shorter REM latency on MSLT, and higher prevalence of SP and HH, and (2) more prominently disrupted nocturnal sleep manifested as lower sleep efficiency, higher arousal index and higher amount of stage N1 on n-PSG compared to the NA w/o CA group consistent with previous reports[6, 33]. This study first assessed the comorbidity rates and degree of SRBD, PLMS, and polysomnographically diagnosable RBD/RWA among large number of Japanese drug naive patients with NA-spectrum disorders. Results revealed that patients with NA-CA had higher rates and degree of PLMS and RBD/RWA compared with those with NA w/o CA or IHS w/o LST. In terms of PLMS, 24.7% of patients with NA-CA showed 5/h^-1^ or more of total PLMS index; the percentage was more than twice as high as that in patients with NA w/o CA or IHS w/o LST. They also showed a higher comorbidity rate (10.2%) of patients with 15/h^-1^ or more of total PLMS index than the other two patients groups. In previous studies, the rate of patients having more than 5/h^-1^ of PLMS index was higher than 60%[9, 10, 34]. The rate with more than 15/h^-1^ of PLMS index was 25.0–50.0% in patients with NA-CA[11, 35]. Those rates were much higher than those shown by the present Japanese subjects. The reasons for the discrepancy are inferred as the followings. First, considering that racial differences in the prevalence of RLS and PLMS are partially attributable to a certain difference in genetic background[26, 36], genetic differences might also explain the lower prevalence of PLMS in the Japanese patients with NA-CA. Secondly, the subjects examined in the present study were much younger than those in earlier reports despite PLMS is more likely to occur in middle-aged or older persons even in patients with narcolepsy[35]. As for PLMS index, patients with NA-CA showed the highest PLMS index consistent with the previous studies[9, 11, 12, 37]. Abnormal dopaminergic function is known to play an important role on the mechanism of PLMS[38]. Considering that hypocretin neuron mediates dopaminergic function[39], the order of PLMS index; NA-CA>NA w/o CA> IHS w/o LST might reflect hypocretin level in cerebrospinal fluid[4, 20, 21].

Polysomnographically diagnosable RBD was found in 1.9% of patients with NA-CA, 0.3% of NA w/o CA and none of IHS w/o LST. Previous studies, which recruited narcoleptics around 40 yrs of age including those under treatment and questioned thoroughly about the history of RBD, reported the prevalence of RBD much higher than that in the current study; however, they reported similar order of prevalence of RBD; 36.0–70.8% of patients with NA-CA and about 40.0% of those with NA w/o CA[13, 15, 35, 40]. The rate of polysomnographically diagnosable RBD in patients with drug-naïve Japanese patients with NA-CA was almost compatible with the rate of patients with RBD identified only on n-PSG (4.2%) in a previous study[41].

In this study, the rate of patients with RWA finding was highest in NA-CA (26.6%). In spite of the lack of structured clinical interview for diagnosis of RBD, the rate was much higher than that of polysomnographically diagnosable RBD (1.9%). Considering that patients with narcolepsy are likely to show mild and elementary movements manifested on EMG activity rather than complex movements[13, 15], oversight of polysomnographic RBD manifestation could be lead in these patients. However, the patients with narcolepsy showing isolated finding of RWA are worth considering to have possibility to develop clinical RBD in the future[42]. Both tonic and phasic EMG activity were highest in patients with NA-CA among the three groups consistent with previous studies where narcoleptics had a greater amount of RWA compared to IHS[12, 16]. A recent study has suggested hypocretin-1 deficiency as a predictor of higher muscle activity during REM sleep and RBD symptoms[40]. Other reports have shown that a missing excitatory pathway of hypocretin neurons to sublaterodorsal tegmental nucleus, one of regional areas inferred to be associated with RWA, might be associated with the formation of RWA in narcolepsy[43]. Considering these observations, CSF-hypocretin depletion associated with the presence of HLA-DQB1*0602[4] could be explainable for the current results of quantity of RWA as well as PLMS in the NA-spectrum disorders.

Previous reports have described a relatively higher prevalence of SRBD in narcolepsy, which is mainly due to obesity caused by altered energy homeostasis in relation to hypocretin-1 deficiency[17–19, 21]. In the present study, the prevalence was lower compared to those reported in the previous studies possibly because of the present subjects of younger age. Besides, consistent with a previous report[37], the present study showed no differences in the rates of patients with OSA or CSA, or degree of the disorder among the patient groups of narcolepsy-spectrum disorders. This discrepancy could not be likely to come from significant difference in the grade of obesity or age among the three groups because the comparison of either BMI or age among the three groups showed small effect size. Another possible reason is that an attenuated ventilatory response to hypercapnia or hypoxia due to a loss of hypocretin-1 [23] lengthens the duration of apneic episodes and results in a decrease in the number of apneic episodes. A further study is necessary to compare the duration of each type of apneic episodes or time with oxygen desaturation as well as the number of respiratory events as degree of SRBD among the NA-spectrum disorders.

Several limitations of this study should be noted. First, we could neither measure the hypocretin-1 levels in cerebrospinal fluid (CSF) of these subjects nor determine the positivity for HLA-DRB1*1501/DQB1*0602. Further studies must be undertaken to clarify the relations among the degree of the motor dyscontrol, HLA-positivity and CSF hypocretin-1 levels in the NA-spectrum disorders classified according to the ICSD-3[44]. Second, no structured interview was conducted to evaluate RBD at the first visit and amount of RWA could not be compared to a recommended optimal cut-off value for diagnosis of RBD in the ICSD-3[45]. Unfortunately, we had not recorded EMG activity in the flexor tensor digitorum during the period in which the current subjects undertook n-PSG and had scored RWA according to the AASM criteria. As for the scoring methods of RWA proposed by the AASM, no optimal cut-off value for diagnosis of RBD had been reported during the study period. Therefore, we adopted the cut-off value for phasic EMG activity in the submentalis muscle referring to a recent report in which phasic EMG activity in the submentalis muscle was scored in a similar way to the AASM scoring methods[32]. Third is the possible heterogeneity of the patients diagnosed as IHS w/o LST. The subjects categorized in IHS w/o LST having sleep apnea or PLMS with EDS can be classified as SRBD or PLM disorder not as IHS w/o LST because it was unclear whether their EDS is due to the respiratory events/movements during sleep or to idiopathic hypersomnia. In addition, in this study, frequency of NA w/o CA or IHS w/o LST in our consecutive patients was quite higher than that of NA-CA inconsistent with previous reports[46, 47]. The reason for this observation is unclear, however, unignorable number of patients with other sleep disorders, namely, differential diagnosis of NA w/o CA or IHS w/o LST such as behaviorally induced insufficient sleep syndrome or circadian rhythm sleep disorders could be included in the groups of NA w/o CA or IHS w/o LST, because usual sleep-wake patterns were not checked using sleep log or actigraphy for all the subject patients. Fourth, the technologists could not score the n-PSG data blinded to potential clinical diagnosis because they scored them as clinical routine.

## Conclusions

Japanese drug naïve patients with NA-CA showed higher comorbidity rates and degree of PLMS and polysomnographically diagnosable RBD with excessive motor activity compared to those with NA w/o CA or IHS w/o LST; the rates were lower than those for Western patients.
